# Supplementary material for: Drosophila macrophages switch to aerobic glycolysis to mount effective antibacterial defense
Source: eLife. 2019 Oct 14;8:e50414. doi: 10.7554/eLife.50414 (PMC6867711; doi:10.7554/eLife.50414)
Supplement: Transparent reporting form [file elife-50414-transrepform.docx]

***eLife’s* transparent reporting form**

We encourage authors to provide detailed information *within their submission* to facilitate the interpretation and replication of experiments. Authors can upload supporting documentation to indicate the use of appropriate reporting guidelines for health-related research (see [EQUATOR Network](http://www.equator-network.org/%20)), life science research (see the [BioSharing Information Resource](https://biosharing.org/)), or the [ARRIVE guidelines](http://www.plosbiology.org/article/info:doi/10.1371/journal.pbio.1000412) for reporting work involving animal research. Where applicable, authors should refer to any relevant reporting standards documents in this form.

If you have any questions, please consult our Journal Policies and/or contact us: [editorial@elifesciences.org](mailto:editorial@elifesciences.org).

**Sample-size estimation**

- You should state whether an appropriate sample size was computed when the study was being designed
- You should state the statistical method of sample size computation and any required assumptions
- If no explicit power analysis was used, you should describe how you decided what sample (replicate) size (number) to use

Please outline where this information can be found within the submission (e.g., sections or figure legends), or explain why this information doesn’t apply to your submission:

The size of the sample used in the experiment was estimated based on our previous experience with the experimental model.

For experiments characterizing individuals resistance to bacterial infection, thousands of individual per genotype were used. In exacting and expensive experiments such as FACS followed enzymatic activity measurements, the number of replicates was limited.

In all cases the raw data are displayed in figures, so number of biological replicates can be seen directly from plots.

The information about biological and technical replicates used is provided in parts Figure legends, and material and methods.

**Replicates**

- You should report how often each experiment was performed
- You should include a definition of biological versus technical replication
- The data obtained should be provided and sufficient information should be provided to indicate the number of independent biological and/or technical replicates
- If you encountered any outliers, you should describe how these were handled
- Criteria for exclusion/inclusion of data should be clearly stated
- High-throughput sequence data should be uploaded before submission, with a private link for reviewers provided (these are available from both GEO and ArrayExpress)

Please outline where this information can be found within the submission (e.g., sections or figure legends), or explain why this information doesn’t apply to your submission:

In all experiment minimally three biological replicates were used.

Since many experiments are based on crossed fly lines, as a biological replicate was considered crossing of parental lines and selection of progeny.

In technically exacting methods, technical triplicates were used to uncover potential bias cased by technical problems during measurements.

In experiments with live individuals, the technical replicates were made by dividing the individuals into several vial, to avoid overcrowding, and affecting of outcome of experiment for unexpected reasons.

No data were excluded from statistical analyses.

Compared fly lines were always carrying as much identical genetic background as possible.

**Statistical reporting**

- Statistical analysis methods should be described and justified
- Raw data should be presented in figures whenever informative to do so (typically when N per group is less than 10)
- For each experiment, you should identify the statistical tests used, exact values of N, definitions of center, methods of multiple test correction, and dispersion and precision measures (e.g., mean, median, SD, SEM, confidence intervals; and, for the major substantive results, a measure of effect size (e.g., Pearson's r, Cohen's d)
- Report exact p-values wherever possible alongside the summary statistics and 95% confidence intervals. These should be reported for all key questions and not only when the p-value is less than 0.05.

Please outline where this information can be found within the submission (e.g., sections or figure legends), or explain why this information doesn’t apply to your submission:

All statistical test were made in Graph Pad Prism. Only standard test were used and mean and standard deviation is displayed in plots. Statistical correction for multiple tests were used.

Al the necessary details about statistics and data analyses are mentioned in sections Figure legends, and Material and methods.

(For large datasets, or papers with a very large number of statistical tests, you may upload a single table file with tests, Ns, etc., with reference to sections in the manuscript.)

**Group allocation**

- Indicate how samples were allocated into experimental groups (in the case of clinical studies, please specify allocation to treatment method); if randomization was used, please also state if restricted randomization was applied
- Indicate if masking was used during group allocation, data collection and/or data analysis

Please outline where this information can be found within the submission (e.g., sections or figure legends), or explain why this information doesn’t apply to your submission:

For most of the experiments only males were used to avoid complications with female reproductive cycle.

In cases that the individuals of the same genotype were split, the allocation into experimental group has been done randomly.

No masking during experimental procedures and data analyses was used.

All necessary information are mentioned in sections Figure legends, and Material and methods.

**Additional data files (“source data”)**

- We encourage you to upload relevant additional data files, such as numerical data that are represented as a graph in a figure, or as a summary table
- Where provided, these should be in the most useful format, and they can be uploaded as “Source data” files linked to a main figure or table
- Include model definition files including the full list of parameters used
- Include code used for data analysis (e.g., R, MatLab)
- Avoid stating that data files are “available upon request”

Please indicate the figures or tables for which source data files have been provided:

In all figures, original data are displayed, while each spot represents unique number used for statistical analyses. The raw numerical data for all plots are provided as a Source numerical data associated with particular plot. Further recalculation and data meta-analyses is thus possible. All necessary information are mentioned in sections Figure legends, and Material and methods.
